# Functional activity and tumor-specific expression of dual oxidase 2 in pancreatic cancer cells and human malignancies characterized with a novel monoclonal antibody

**DOI:** 10.3892/ijo.2013.1821

**Published:** 2013-02-11

**Authors:** YONGHZONG WU, SMITHA ANTONY, STEPHEN M. HEWITT, GUOJIAN JIANG, SHERRY X. YANG, JENNIFER L. MEITZLER, AGNES JUHASZ, JIAMO LU, HAN LIU, JAMES H. DOROSHOW, KRISHNENDU ROY

**Affiliations:** 1 Laboratories of Molecular Pharmacology, National Cancer Institute, National Institutes of Health, Bethesda, MD 20892, USA; 2 Pathology, Center for Cancer Research, National Cancer Institute, National Institutes of Health, Bethesda, MD 20892, USA; 3 Division of Cancer Treatment and Diagnosis, National Cancer Institute, National Institutes of Health, Bethesda, MD 20892, USA

**Keywords:** dual oxidase, NADPH oxidase, reactive oxygen species, pancreatic cancer, gene expression

## Abstract

Dual oxidase 2 (Duox2), one of the seven members of the NADPH oxidase gene family, plays a critical role in generating H_2_O_2_ for thyroid hormone biosynthesis and as an integral part of the host defense system of the respiratory epithelium and the gastrointestinal tract. Recent evidence suggests that the regulation of Duox2 expression is under the control of pro-inflammatory cytokines and that Duox2-induced reactive oxygen species (ROS) contribute to the inflammation-related tissue injury that occurs in two pre-malignant, inflammatory conditions: chronic pancreatitis and inflammatory bowel disease. Because no reliable Duox antibodies are commercially available, we report the development of a murine monoclonal antibody (MAb) to Duox2 (clone Duox S-12) and its use for the characterization of Duox2 expression in human tumors, tumor cell lines and normal tissues. Duox S-12 specifically detected both endogenously- and ectopically-expressed Duox2 protein by immunoblotting, immunofluorescence microscopy and immunohistochemistry (where both membranous and cytoplasmic staining were present). Duox2 expression detected by Duox S-12 was functionally coupled to the generation of H_2_O_2_ in pancreatic cancer cells that expressed Duox2 and its cognate maturation factor DuoxA2. Although Duox S-12 recognizes ectopically expressed Duox1 protein because of the extensive amino acid homology between Duox1 and Duox2, the lack of substantial Duox1 mRNA expression in human tumors (except thyroid cancer) allowed us to evaluate Duox2 expression across a wide range of normal and malignant tissues by immunohistochemistry. Duox2 was expressed at elevated levels in many human cancers, most notably tumors of the prostate, lung, colon and breast while brain tumors and lymphomas demonstrated the lowest frequency of expression. The Duox-specific monoclonal antibody described here provides a promising tool for the further examination of the role of Duox-dependent reactive oxygen production in inflammation-related carcinogenesis, where alterations in oxidant tone play a critical role in cell growth and proliferation.

## Introduction

The first demonstration that tumor cells could generate ROS at rates that approach the capacity of phagocytic leukocytes occurred over two decades ago (1,2). At that time, it was appreciated that oxygen radical generation by tumor cells might contribute to invasion and metastasis, as well as the development of ROS-related DNA damage (2–4). However, a complete understanding of the sources of tumor cell ROS has only recently begun to be developed, having awaited the discovery over the past decade of the family of six epithelial NADPH oxidases (Noxs) that have significant homology with the membrane oxidase of leukocytes (5), and the development of reagents that allow evaluation of expression of the members of the Nox gene family across different tissues and tumors. Recent evidence suggests that some NADPH oxidases may play a critical role in enhancing tumor cell proliferation and angiogenesis across a broad range of histological subtypes of malignancy (6,7).

Dual oxidase 2 (Duox2) is one member of the epithelial Nox family that generates H_2_O_2_ in the service of several critical physiological functions, including thyroid hormone biosynthesis and host defense (8,9). It has two catalytic sites: an NADPH oxidase as well as a heme peroxidase that function to generate extracellular H_2_O_2_(10). Duox2 is one of two closely-related Nox isoforms, the other being Duox1, that share greater than 85% homology at the amino acid level (11); the membrane-spanning regions of these proteins are highly homologous to the gp91phox domain of the phagocytic oxidase (Nox2). The N-terminal heme peroxidase-like extracellular domain is also related to other peroxidases that convert O_2_•^−^ to H_2_O_2_. In addition to the NADPH oxidase and peroxidase-like domains, two cytosolic, calcium-binding EF-hand domains have been described which may explain the requirement for the presence of micromolar calcium concentrations to generate functional oxidase activity. Finally, it has recently become clear that reactive oxygen formation *in vivo* requires the presence in cells of a dual oxidase maturation factor (DuoxA2), an ER-resident protein that is necessary for post-translational processing and translocation of an enzymatically functional Duox2 complex to the plasma membrane (12).

Duox2 has also been implicated in the pathogenesis of chronic inflammatory, pre-neoplastic conditions, such as inflammatory bowel disease and chronic pancreatitis (13–15). In the case of inflammatory bowel disease, the expression of Duox2 is significantly increased in human colon biopsies, and in isolated intestinal epithelial cells, from patients with both Crohn’s disease and ulcerative colitis compared to expression levels in normal adjacent colonic mucosa, suggesting that an unchecked ROS response to pathogens could contribute to the tissue injury observed in these chronic inflammatory disorders (13). These results are consistent with the observation that the expression of Duox2 is upregulated 10-fold in pre-malignant adenomatous polyps of the colon compared to adjacent colonic mucosa as determined by expression array analysis (16), as well as our finding that Duox2 expression at the mRNA level is dramatically increased in some surgically-resected colon cancers (7).

Unfortunately, although certain physiological functions of Duox2 are known in detail, such as its role in thyroid hormone biosynthesis, immunochemical detection studies of Duox2 that could have important clinical implications remain to be initiated because of a lack of specific Duox2 antibodies. The expression of Duox2 at the protein level in human tumors or in pre-malignant conditions is, therefore, effectively unknown, as well as its relative intracellular localization in specific tissues both normal and malignant. Only a small number of studies have been performed that have attempted to examine Duox2 expression in human tissues by immunohistochemical techniques; in some of these studies, antisera were prepared against a short stretch of a Duox2 peptide that might make establishing specificity difficult (17). Currently-available polyclonal antibodies used to detect Duox2 have been developed without always identifying the initiating antigen or establishing specificity by genetic means, western blot analysis or immunohistochemistry. Hence, we chose to develop a Duox2 monoclonal antibody that would be applicable to a variety of investigative applications in clinical specimens so that a full characterization of Duox2 expression in normal as well tumor tissues would be possible.

Herein we report the production and characterization of a high quality monoclonal antibody that appears to be specific for the detection of functional Duox protein and that can be used effectively for many immunochemical applications. We have utilized this antibody to evaluate the expression of Duox in both normal tissues and in a variety of human tumors by tissue microarray. Our results demonstrate for the first time that Duox protein is highly overexpressed in cancers of the prostate, lung, colon and breast compared to normal tissues from these organs; and that, in contrast, Duox protein is not found in abundance in non-Hodgkin lymphomas or glioblastoma multiforme.

## Materials and methods

### Materials

Recombinant human IFN-γ (catalog no. 285-IF) was purchased from R&D Systems. Antibody against human β-actin (catalog no. A3853) was acquired from Sigma-Aldrich. Human Duox2 primer (catalog no. Hs00204187_m1), human Duox1 (catalog no. Hs00213694), human β-actin (catalog no. Hs99999903_m1), and TaqMan Universal PCR mix (catalog no. 4364340) were purchased from Applied Biosystems.

### Cell culture

The human pancreatic cancer cell lines BxPC-3 (catalog no. CRL-1687), MIA PaCa-2 (catalog no. CRL-1420™), and PANC-1 (catalog no. CRL-1469™) were obtained from the American Type Culture Collection (Manassas, VA). BxPC-3 cells were cultured in RPMI-1640 medium (catalog no. SH30255.01; HyClone) with 1% pyruvate and 10% FBS. MIA PaCa-2 cells were cultured in Dulbecco’s modified Eagle’s medium with 10% FBS and horse serum to a final concentration of 2.5%. PANC-1 cells were cultured in Dulbecco’s modified Eagle’s medium with 10% FBS. Cells were cultured in a humidified incubator at 37°C in an atmosphere of 5% CO_2_ in air.

### Cloning, expression and purification of a partial recombinant Duox2 protein

**Figure 1:**
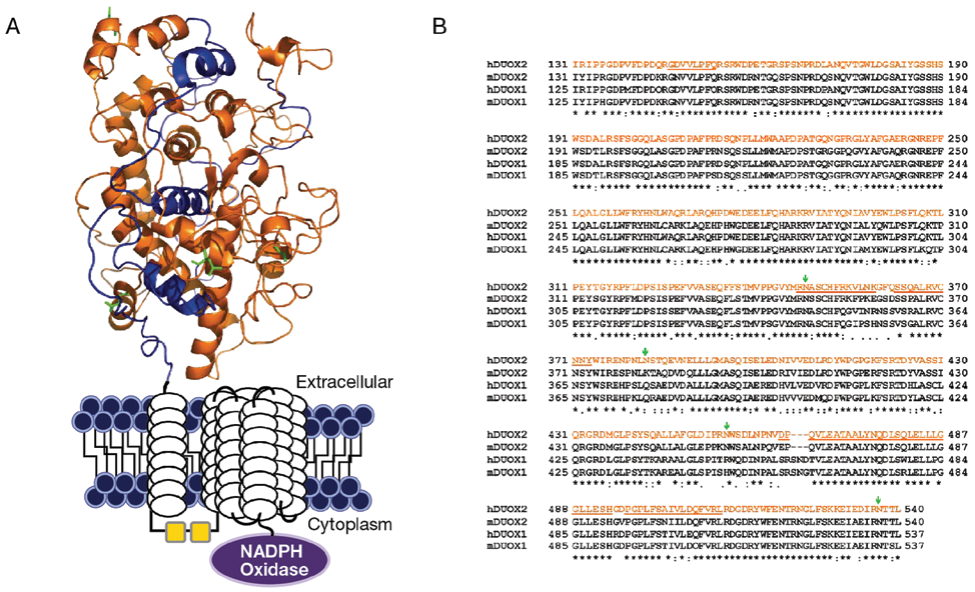
Sequence and structural analysis of the antigen used to develop the hDuox2 MAb. (A) Schematic view of the structural features of the hDuox2 protein. Each hDuox protein contains an N-terminal peroxidase-like region, 7 putative TM domains (white cylindrical loops), 2 EF calcium binding sites (yellow squares) and an NADPH oxidase domain (purple). The peroxidase-like domain (hDuox21-599) structural model was produced by the SWISS-MODEL program server and Pymol. The orange highlighted segment represents the antigen expressed for antibody development (AA131–540). Amino acids displayed as sticks (green) are predicted to be glycosylated by NetNGlyc 1.0 Server (N348, N382, N455, N537). (B) Sequence alignment of the hDuox2 amino acid region expressed for antigen development. hDuox2 amino acids 131–540 (highlighted in orange) were aligned against both human and mouse isoforms; h, *H. sapiens*; m, *M. musculus*. Underlined amino acid regions of highest antigenicity were predicted by EMBOSS Antigenic and predicted sites of N-glycosylation are indicated by green arrows.

To generate a monoclonal antibody specific for Duox2, the human Duox2 protein sequence was obtained from the NCBI data base; it contains 1,548 amino acids of an integral membrane glycoprotein. Initially, we were unsuccessful in expressing the full length Duox2 protein in BL21 (DE3) *E. coli* utilizing different plasmid vector backbones (data not shown). After a careful bioinformatics approach to total protein structure, we identified the amino terminal end of the Duox2 sequence that represents 410 amino acids (NH2 terminal 131–540 amino acid peptides) to be a sequence of high potential immunogenicity for antibody production (data not shown). Using a human full length Duox-cDNA plasmid as template, through PCR, a 1,230 BP fragment corresponding to a 131–540 amino acid sequence was amplified and sub-cloned into a PET30a(+) vector. The NH2 terminal 131–540 (410) amino acids represent a unique peroxidase-like domain region of the Duox2 sequence that we felt would be suitable for antibody production (Fig. 1). Prior to expression of the pET30a(+)-DUOX2-410AA, the nucleotide sequence of the entire gene construct was re-sequenced; we found that our desired sequence was in the right order. Expression of Duox2-410AA was detected in the culture after 2 h of induction with IPTG by analysis of the SDS-PAGE bacterial pellet, where the appearance of a ∼45-kDa band indicated the synthesis of the recombinant Duox2-410AA-His-Tag. Soluble cytosolic fractions of induced BL21 lysate demonstrated that most of the induced protein band was found in the soluble fraction, as confirmed by SDS-PAGE (data not shown). The truncated Duox2-410AA protein was purified to near homogeneity using Ni-NTA sepharose resin as evaluated by SDS-PAGE (data not shown). From 1 liter of *E. coli* culture, we purified 5.0 mg protein with an apparent purity of 98% as revealed by Coomassie Blue staining. The recovery and purification fold were greater than 80% (data not shown).

### Production of monoclonal antibodies

To generate monoclonal antibodies, four Balb/c mice were immunized subcutaneously with a fusion protein containing 50 *μ*g Duox2-410AA-His-Tag antigen in complete Freund’s adjuvant (Sigma, Gillingham, UK); following this protocol, approximately 200 *μ*g of protein was injected per animal over ten weeks. This program was followed by three further subcutaneous booster immunizations of 50 *μ*g Duox2-410AA-His-Tag antigen in incomplete Freund’s adjuvant (Gibco-BRL, Grand Island, NY) at 15-day intervals. Seven days after the final booster immunization, test bleeds were taken from each mouse, and the resulting serum samples (test sera) were screened alongside the corresponding pre-immune serum sample from each mouse for antibody binding to Duox2-410AA-His-Tag antigen. Ninety-six-well micro-titer plates were used for the HRP-conjugate enzyme-linked immunosorbent assay to screen pre- and post-immune sera (data not shown).

### Panel of monoclonal antibodies against Duox2

According to standard procedures, splenocytes from the immunized mouse were fused with SP2/0 mouse myeloma cells at a ratio of 10:1 using a conventional polyethylene glycol (PEG) 1500 (Sigma) fusion protocol, and the resulting hybridomas were selected in HAT medium. Cell culture supernatants of the hybrid cell colonies were screened for antibodies by ELISA, and positive cell lines were subcloned three times by limiting dilution. Finally, ELISA titer results were calculated as the mean absorbance at 450 nm for each serial dilution of the test and pre-immune serum samples corrected by subtracting the blank mean absorbance at 450 nm (mean absorbance 450 nm for non-specific binding of the anti-mouse Ig polyvalent HRP conjugate to Duox2-410AA-His-Tag coated wells) (data not shown). Hybridoma cell lines producing anti-Duox2-410AA-His-Tag antibodies were cloned from single cells, expanded and cryo-preserved according to standard procedures. Additionally, the hybridomas were further screened to identify antibodies reacting with both Duox2-410AA-His-Tag antigens. The experimental screenings with Duox2-410AA-His-Tag coated on ELISA plates selected several monoclonal antibodies; those that reacted to the His-tag were discarded. Many hybridoma clones were initially identified (47 clones) as producing anti-Duox2-410AA-His-Tag antibodies; 34 clones reacted only with truncated Duox2-410AA. During further passage in tissue culture, 10 of these 34 clones either died or stopped producing antibody. We successfully developed and cryo-preserved 24 stable anti-Duox2-410AA producing hybridoma cell lines (data not shown).

### Development of MIA PaCa-2 cells stably transfected with Duox2 cDNA

MIA PaCa-2 cells were transfected with an HA-tagged full length human Duox2 gene in a CMV driven expression vector (pcDNA3.1) using the Lonza transfection protocol in 100 *μ*l of transfection buffer (Amaxa Cell line Nucleofector Kit V) (Program: T-027) utilizing the Amaxa Nucleofector Device (Lonza, ME). Stable clones of MIA PaCa-2 cells with empty vectors as well as those expressing the HA-tag-Duox2 were developed by selection in G418. Because the production of H_2_O_2_ by the Duox2 complex requires the presence of both Duox2 and its maturation factor (DuoxA2) (12), the HA-tag-Duox2 stable MIA PaCa-2 clonal cells were further transiently transfected with the human full length DuoxA2 gene in a mammalian expression vector (pcDNA3.1) to evaluate functional enzymatic activity.

### Transient transfection of COS-7 cells

Transfection of COS-7 cells was performed according to the manufacturer’s instructions using the Lonza transfection protocol and transfection reagent in 100 *μ*l of transfection buffer (Amaxa Cell line Nucleofector Kit R) (program: A-024) utilizing the Amaxa Nucleofector Device (Lonza, Rockland, ME). For each transfection, 2 *μ*g of plasmid DNA (pcDNA3.1/HA-Duox1 or HA-Duox2 or empty vector) was used. After 48 h of incubation, cells were lysed and analyzed for RNA and protein content.

### RNA extraction, cDNA synthesis and quantitative real-time RT-PCR assay

Total RNA was extracted with the RNeasy mini kit (catalog no. 74104; Qiagen) according to the manufacturer’s instructions. Two micrograms of total RNA was used for cDNA synthesis, using SuperScript II reverse transcriptase (catalog no. 18080-044) and random primers (catalog no. 48190-011; Invitrogen) in a 20 *μ*l reaction system, with the following cycles: 25°C for 5 min, 42°C for 50 min and 75°C for 5 min. After the reaction was complete, the RT-PCR products were diluted with diethylpyrocarbonate/H_2_O to 100 *μ*l for real-time PCR. Real-time RT-PCR was performed in 384-well plates in a 20 *μ*l reaction system containing 2 *μ*l of diluted cDNA, 1 *μ*l of primer mixture, 7 *μ*l of H_2_O, and 10 *μ*l of TaqMan 2X reaction mixture. PCR was carried out under default cycling conditions, and fluorescence was detected with the ABI 7900HT Sequence Detection System (Applied Biosystems, Foster City, CA). Triplicate determinations were performed for each sample that was used for real-time PCR; the mean value was calculated and the data in the final figures represent the results of three independent experiments. Relative gene expression was calculated as the ratio of the target gene to the internal reference gene (β-actin) multiplied by 10^3^ based on C_t_ values.

### Western blot analysis

For preparation of whole-cell extracts, cell pellets from BxPC-3, MIA PaCa-2, and PANC-1 cells, treated with or without IFN-γ, were lysed with 1X RIPA lysis buffer (catalog no. 20–188; Millipore, Temecula, CA), with the addition of a phosphatase inhibitor tablet (catalog no. 04-906-837001; Roche) and a protease inhibitor tablet (catalog no. 11-836-153001; Roche). The protein concentrations of whole-cell extracts were measured by using the BCA Protein Assay Kit (Pierce). Cell extracts were mixed with an equal volume of 2X SDS protein gel loading buffer (catalog no. 351-082-661; Quality Biological); and when required, the samples were denatured by heating at 95°C for 5 min. A total of 50 *μ*g of whole-cell extract was loaded onto a 4–20% Tris glycine gel (catalog no. EC6028; Invitrogen), and the proteins were separated and electrophoretically transferred to nitro-cellulose membranes using I Blot gel transfer stacks (catalog no. IB 3010-01; Invitrogen). The membranes were blocked in 1X TBST buffer with 5% non-fat milk for 1 h at room temperature and then incubated with primary antibody overnight in TBST buffer. Membranes were washed three times in 1X TBST buffer and incubated with HRP-conjugated secondary antibody for 1 h at room temperature with shaking. The antigen-antibody complex was visualized with SuperSignal West Pico Luminol/Enhancer Solution (catalog no. 1856136, Thermo Scientific). Final characterization and evaluation of Duox2 protein expression was determined from the whole-cell extract, mixed with an equal volume of 2X SDS loading buffer but without boiling. For the analysis of proteins other than Duox2, the mixture of cell extract with loading buffer was boiled for 5 min. Although the pancreatic cancer cell lines utilized for these experiments do not contain measurable Duox1 mRNA, because our antibody cross-reacts with Duox1, we have referred to the protein it detects as ‘Duox’.

### Extracellular H_2_O_2_ measurement using Amplex Red^®^

The Amplex Red^®^ Hydrogen Peroxide/Peroxidase Assay Kit (catalog no. A22188; Invitrogen) was used to detect extracellular H_2_O_2_ release. MIA PaCa-2 cells stably expressing Duox2 were transiently transfected with DuoxA2; 48 h following transient transfection with DuoxA2, extracellular H_2_O_2_ release was measured. In preparation for determination of H_2_O_2_ release, MIA PaCa-2 cells were washed twice with 1X PBS, trypsinized and dispersed thoroughly. Cells were counted to produce a 20-*μ*l cell suspension containing 2×10^4^ live cells in 1X Krebs-Ringer phosphate glucose (KRPG) buffer. The cells were mixed with 100 *μ*l of Amplex Red reagent containing 50 *μ*M Amplex Red and 0.1 units of HRP per ml in KRPG buffer with or without 1 *μ*M ionomycin and incubated at 37°C for 60 min. The fluorescence of the oxidized 10-acetyl-3,7-dihydroxyphenoxazine was measured at an excitation wavelength of 530 nm and an emission wavelength of 590 nM, using a SpectraMax Multiplate reader (Molecular Devices, Sunnyvale, CA). H_2_O_2_ was quantified with an H_2_O_2_ standard curve over a concentration range from 0 to 2 *μ*M. Each value in the figure represents a mean of quadruplicate samples from 16 readings.

### Immunofluorescence microscopy analysis of HA-Duox2-expressing MIA PaCa-2 cells

MIA PaCa-2 cells (1×10^5^) stably-transfected with HA-tagged human full length Duox2 cDNA were plated in 4-well glass slides (Lab-TekII Chamber slide, Cat no. 154526, Thomas Scientific, Swedesboro, NJ) containing 1.0 ml of complete growth medium. Cells in the slide chamber were grown overnight, then were fixed with cold methanol at −20°C for 5 min and washed once with 1X PBS, pH 7.4. Non-specific binding of proteins to the section was reduced (blocked) with 0.1% Triton in 1X PBS containing 5% BSA for 60 min at room temperature (RT). After 1 h, the medium was aspirated and the sections incubated for an additional 60 min with the primary antibody (either an anti-HA monoclonal or the Duox S-12 monoclonal antibody) reconstituted in 1X PBS + 5% BSA in a dilution of 1:100 and 1:500 respectively. Next, the slides were washed three times for 3 min with 1X PBS and then incubated for an additional 60 min with 1X PBS + 5% BSA containing the secondary antibody conjugated with fluorescein isothiocyanate (FITC); anti-rat for the HA antibody or anti-mouse for the Duox S-12 monoclonal antibody (1:200, Jackson Immune Research Laboratories). The sections were washed again as above with 1X PBS + 5% BSA and followed by a short (less than 10 sec) wash with MilliQ water, allowed to air-dry at RT, and mounted in vectashield mounting medium with 4’,6-diamidino-2-phenylindole (DAPI) (Vector Laboratories Inc., Burlingame, CA; Cat no. H-1200) within 1 h. Fluorescence microscopy was performed with a Leica DM 500B fluorescence microscope by selecting the green emission filter for FITC, with an excitation filter transmitting light with a wavelength of 480±40 nm and an emission filter transmitting light with a wavelength of 527±30 nm. The images were viewed with a Leica oil-immersion objective lens (40×). Nuclear counter staining was performed with DAPI, and visualized by selecting a blue filter. The fluorescence excitation maximum for DAPI is 360±40 nm and the emission maximum is 470±40 nm.

### Immunohistochemical staining of Duox2-expressing MIA PaCa-2 cells

Ectopically- and stably-expressed human Duox2 cDNA in MIA PaCa-2 cells was evaluated in sections from formalin-fixed and paraffin-embedded cells using a standardized method. In brief, the mouse monoclonal antibody to Duox2 (Duox S-12) in a dilution of 1:1,000 was applied for 1 h at room temperature to paraffin-embedded MIA PaCa-2 cells transfected with either Duox2 or vector alone. Binding of the primary antibodies to their antigenic sites in sections was amplified using Vectastain Elite avidin-biotin-peroxidase complex kits (Vector Laboratories Inc.). The antigen-antibody reaction sites were visualized using 3,3-diaminobenzidine for 7 min and, subsequently, sections were counterstained with Mayer’s hematoxylin. Negative controls were performed using isotype immunoglobulins appropriate to the primary mouse antibod ies used (Zymed Laboratories, South San Francisco, CA).

### Immunohistochemical analysis of Duox expression in human tumors and normal tissues

Immunohistochemistry was performed on a National Cancer Institute TARP multi-tumor tissue microarray [TMA (MTA3)] with Duox antibody applied at 1:500 dilution, after antigen retrieval with pH 6.0 buffer (Dako) with a pressure cooker for 20 min. The antigen-antibody complex was detected with Envision+ (Dako) and DAB chromagen. Staining was scored as 0, 1, 2 and 3 corresponding to negative, weak, intermediate and strong respectively, and interpreted as negative (0 and 1+) or positive (2+ and 3+).

### Statistical analyses

Two tailed Student’s t-tests and χ^2^ analyses were performed; values of p<0.05 were considered significant.

## Results

### Determination of antibody specificity by western blot analysis

**Figure 2:**
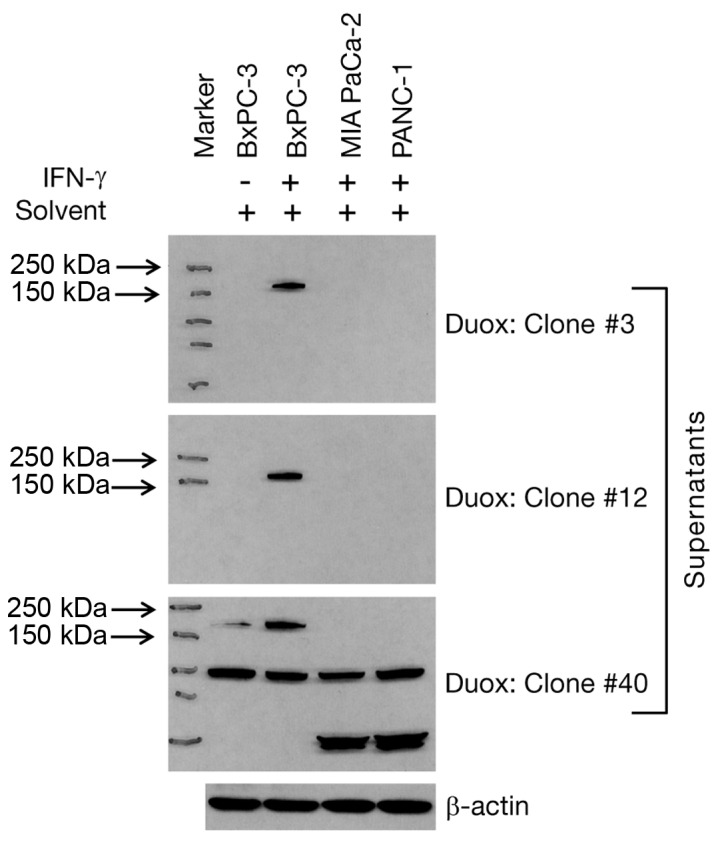
Immunoblot analysis showing reactivity of anti-Duox2 mouse monoclonal hybridoma supernatants S-3, S-12 and S-40. BxPC-3, MIA PaCa-2 and PANC-1 pancreatic cancer cells were treated with 25 ng/ml of IFN-γ or solvent for 24 h. Whole cell lysates were prepared and 50 *μ*g of cell lysate was used for each lane. The Duox2 hybridoma supernatants S-3, S-12 and S-40 were used at 1:1,000 dilutions. β-actin antibody (bottom panel) was used for the protein loading control.

Monoclonal antibodies (denoted S-3, IgG1; S-12, IgG1; and S-40, IgG2b) were selected according to their ELISA immunoreactivity for detailed characterization; specificity was monitored by western blot analysis. The remaining 21 hybridoma cell lines were cryopreserved. Previously, we demonstrated that IFN-γ upregulates the mRNA expression of Duox2 in BxPC-3 human pancreatic cancer cells in a time- and concentration-dependent manner (14). In the same study, we found that Duox2 expression in MIA PaCa-2 and PANC-1 human pancreatic cancer cell lines was unresponsive to IFN-γ exposure. Using supernatants from our three clones (S-3, S-12 and S-40), we performed western blot analysis on IFN-γ-induced, as well as solvent-treated samples of these three cell lines. As shown in Fig. 2, only IFN-γ-treated BxPC-3 cells responded with upregulated Duox2 protein that was detected by all three supernatant antibodies. However, the S-40 clone supernatant demonstrated non-specific protein recognition in all three cell lines, whether or not treatment with IFN-γ was employed. Hence, we did not pursue further studies with the S-40 clone. Because the ELISA affinity for clone S-12 was slightly better than for the supernatant from clone S-3 (0.78 vs. 0.76), we expanded cells from the S-12 clone and have affinity purified the S-12 Duox monoclonal antibody to allow further characterization of its specificity for western blot analysis, immunofluorescence and immunohistochemistry, including tissue microarray analysis.

### Characterization of Duox2 overexpression in MIA PaCa-2 cells

**Figure 3:**
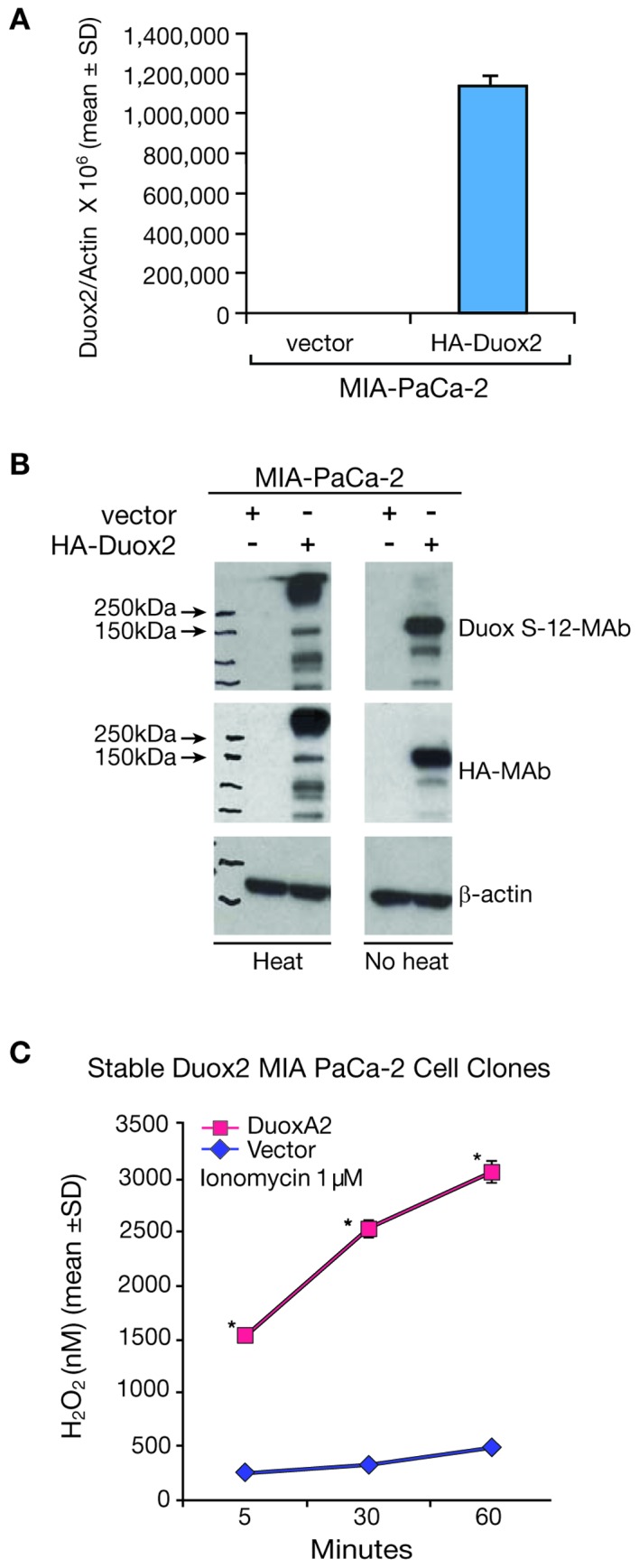
Duox S-12 monoclonal antibody detects exogenous Duox2 protein in MIA PaCa-2 cells with stably transfected human Duox2 cDNA. (A) Quantitative real-time RT-PCR assay of relative Duox2 expression after stable transfection of a human HA-Duox2-pcDNA-3.1 plasmid or empty pcDNA-3.1 vector into MIA PaCa-2 cells, normalized to β-actin. Data represent the mean and standard deviation from triplicate samples. (B) Western blot analysis results from 50 *μ*g of whole cell lysate of MIA PaCa-2 cells stably expressing either empty vector or HA-tagged human Duox2 cDNA. The antibodies used were as follows: upper panel, Duox S-12 MAb; middle panel, HA MAb; and lower panel, β-actin. In the left panel of the figures, cell lysates were reduced and heat denatured at 100°C for 5 min before loading on the gel; in the right panel, the lysates were reduced but not heat-denatured. (C) MIA PaCa-2 cells stably transfected with Duox2 were transiently transfected with a human DuoxA2 cDNA, to evaluate functional oxidase activity as measured by the production of extracellular H_2_O_2_. Human Duox2 expressing MIA PaCa-2 cells were transiently transfected with either empty vector or a human DuoxA2 plasmid. Forty-eight hours after transfection, 2×10^4^ live cells were used for extracellular H_2_O_2_ detection as described in the ‘Materials and methods’. The reaction was conducted in reaction buffer containing cells and 1 *μ*M ionomycin for the indicated times. Hydrogen peroxide levels were calculated using a standard curve of 0–2 *μ*M H_2_O_2_; ^*^p<0.05.

To explore Duox2 expression in additional human tumor cell lines, MIA PaCa-2 pancreatic cancer cells were stably transfected with a full length, human HA-tagged Duox2 cDNA. Duox2 mRNA expression was significantly higher in the Duox2-transfected clone (Fig. 3A) than in the empty vector-transfected clone of MIA PaCa-2 cells. We found by western blot analysis that the Duox S-12 monoclonal antibody selectively recognized cells that overexpressed Duox2 mRNA (Fig. 3B). We found, furthermore, that following heat-denaturation of our tumor cell lysates, Duox2 protein appeared to aggregate as a high molecular weight band, whereas Duox2 protein was recognized at the expected, ∼185 kDa size in non-heat denatured samples from the overexpressing cell line. We made the same observation utilizing an antibody directed against HA (Fig. 3B), where the HA antibody recognized a protein in the heat-denatured sample with a molecular weight above ∼250 kDa. Hence, immunoblots of the same cell extracts demonstrated that the Duox S-12 monoclonal antibody specifically recognized human Duox2 as a unique protein that was of the same size (∼185 kDa) as the protein recognized by the HA-tag antibody. Pre-immune serum exhibited negligible background staining in the whole transferred blot (data not shown). These results reinforce the previous immunoblots (Fig. 2) demonstrating that IFN-γ enhances Duox2 expression in crude cell extracts from BxPC-3 pancreatic cancer cells; in those experiments, IFN-γ upregulated the mRNA expression of Duox2, but not Duox1 or any other member of the NADPH oxidase gene family (data not shown).

### Expression of Duox2 and DuoxA2 leads to H_2_O_2_ production in MIA PaCa-2 cells

To demonstrate the functional activity of Duox2 in MIA PaCa-2 cells, the stably-transfected, Duox2 MIA PaCa-2 cell clones were transiently transfected with a DuoxA2 cDNA or an empty vector; ionomycin-enhanced production of H_2_O_2_ was then examined using the Amplex Red^®^ reagent. As shown in Fig. 3C, MIA PaCa-2 cells stably expressing Duox2 cDNA alone produced minimal amounts of H_2_O_2_; similarly, MIA PaCa-2 cells expressing the empty vector exhibited no significant H_2_O_2_ production, even in presence of the calcium ionophore, ionomycin (data not shown). However, transient expression of DuoxA2 cDNA (which has virtually no constitutive expression in this cell line) in the stably expressing Duox2 MIA PaCa-2 cells resulted in a dramatic increase in H_2_O_2_ production, p<0.05.

### Immunohistochemical analysis of MIA PaCa-2 cells stably transfected with Duox2 cDNA

**Figure 4:**
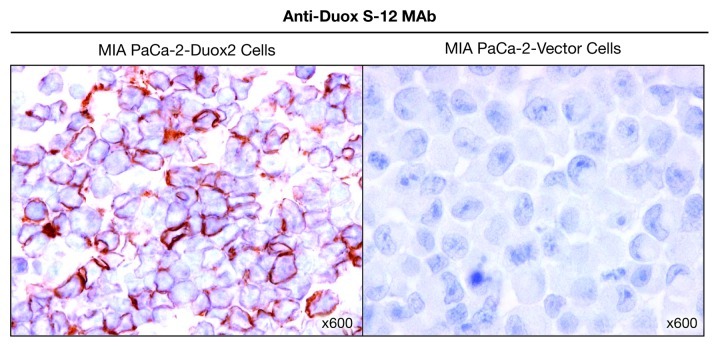
Immunohistological staining of MIA PaCa-2 cells stably transfected with Duox2 using the Duox S-12 MAb. Slides were prepared from paraffin-embedded and formalin-fixed cell pellets of MIA PaCa-2 cells stably transfected either with empty vector (right panel) or with human Duox2 plasmid (left panel). Slides were subjected to immunohistochemical assay using the Duox S-12 MAb as described in ‘Materials and methods’.

We next investigated whether the anti-Duox S-12 monoclonal antibody would be useful for immunohistochemical staining. Using fixed sections of MIA PaCa-2 cells that overexpressed HA-tagged, full length human Duox2, we found negligible background staining when the sections were reacted with pre-immune serum (data not shown). When paraffin-embedded, Duox2-overexpressing MIA PaCa-2 cell pellets were examined with our Duox S-12 antibody (Fig. 4), we found the expected, prominent immuno-staining of the tumor cell plasma membrane. On the other hand, expression of Duox2 protein could not be detected in vector-transfected MIA PaCa-2 cells (Fig. 4).

### Immunofluorescence analysis of MIA PaCa-2 cells expressing Duox2 cDNA

**Figure 5:**
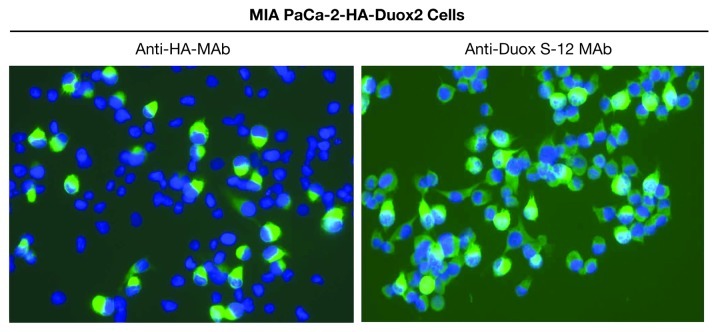
Immunofluorescence assay of MIA PaCa-2 cells overexpressing HA-tagged Duox2 using the Duox S-12 antibody. MIA PaCa-2 cells stably transfected with HA-tagged human Duox2 plasmids were processed for immunofluorescence as described in ‘Materials and methods’. Cells were incubated with HA (left panel) or Duox S-12 monoclonal antibodies (right panel) for 60 min, followed by incubation with FITC-conjugated anti-rat (for HA) or anti-mouse (for Duox2) antibodies for an additional 60 min. Images were obtained with a confocal microscope. Nuclei were counterstained with propidium iodide.

The Duox S-12 monoclonal antibody was further characterized by performing immunofluorescent staining of stably transfected, clonally selected MIA PaCa-2 cells expressing both Duox2 mRNA and an HA tag. Duox2-transfected MIA PaCa-2 cells stained positively with either the anti-HA antibody or the anti-Duox S-12 MAb (Fig. 5). The immunofluorescence pattern for both Duox2 and HA demonstrated intense cytoplasmic staining, with an apparent further enhancement in the plasma and nuclear membranes of some cells. In contrast, cells expressing a control vector did not demonstrate immunofluorescence with either the anti-HA antibody or the anti-Duox S-12 monoclonal antibody (data not shown).

### Duox S-12 monoclonal antibody cross-reacts with human Duox1 protein

**Figure 6:**
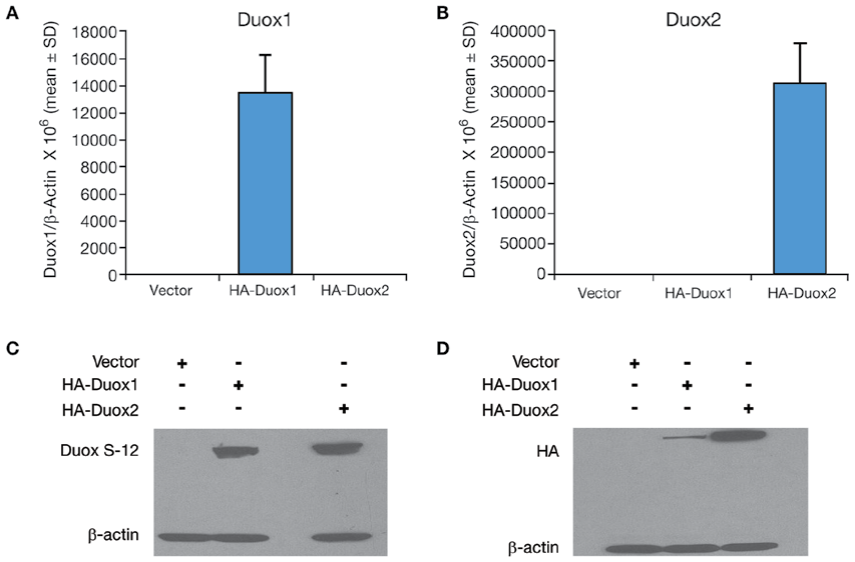
Duox S-12 MAb cross-reacts with Duox1 protein. (A and B) Real-time RT-PCR assay of Duox2 and Duox1 expression were performed in COS-7 cells, after transient transfection with either an empty pcDNA-3.1 vector or an HA-tagged human Duox1 or HA-tagged human Duox2 plasmid cloned into the pcDNA3.1 vector as indicated in the figures. (C and D) Western blot analysis of whole cell lysates prepared from COS-7 cells in the identical situation as described in Fig. 6A and B. Anti-HA MAb was used and is shown in the right panel and Duox S-12 MAb was used for the left panel to detect the overexpressed Duox1 or Duox2 proteins. β-actin antibody was used for the protein loading control.

As shown in Fig. 1, there is significant homology (∼85%) between Duox 2 and Duox1 at the amino terminus of both proteins. This raised the question as to whether our Duox S-12 monoclonal antibody might cross-react with the human Duox1 protein. To resolve this issue, we transiently transfected both HA-tagged human Duox1 and HA-tagged human Duox2 cDNAs into COS-7 cells along with the appropriate vector controls. Analysis by real-time RT-PCR revealed that expression of both Duox1 and Duox2 was detected in COS-7 cells following transient transfection compared to their vector controls (Fig. 6A and B). As demonstrated in Fig. 6C, our Duox S-12 monoclonal antibody recognized both Duox2 and Duox1 proteins. This observation is supported by the western blot analysis shown in Fig. 6D, where an antibody against HA recognized both human Duox1 and Duox2, since both cDNAs were tagged with HA. Thus, we describe our newly-characterized antibody as Duox S-12 because it detects both Duox1 and 2 proteins.

### Expression of Duox in human tumors and normal tissues

**Figure 7:**
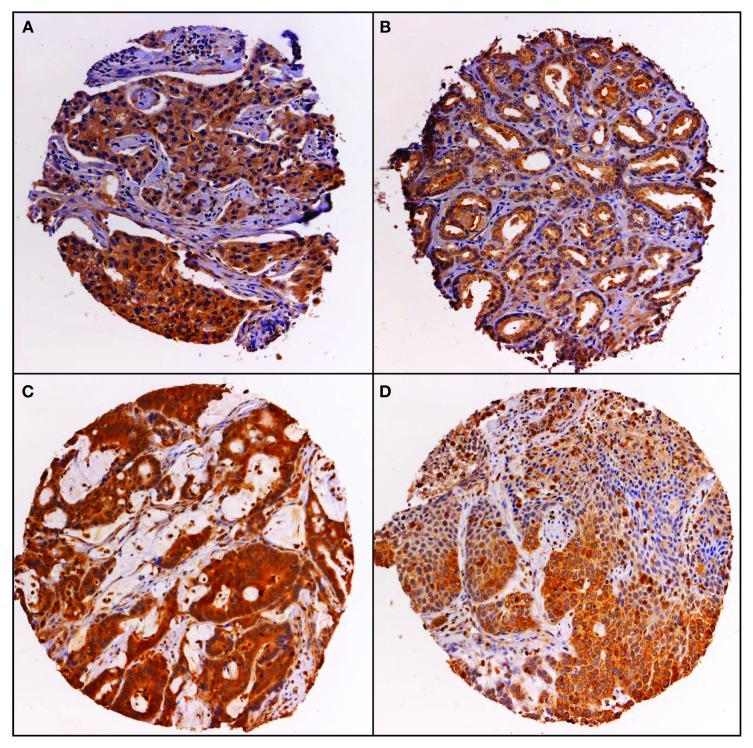
Immunohistochemistry for Duox performed on a multi-tumor tissue microarray. (A) Breast adenocarcinoma, (B) prostate adenocarcinoma, (C) colon adenocarcinoma and (D) squamous cell carcinoma of the lung. All images taken at ×160 magnification.

Expression of Duox was studied by immunohistochemistry on a multi-tumor tissue microarray (TARP MTA3) containing 217 analyzable tumor samples and a sampling of normal tissue. Duox stains in a cytoplasmic pattern, with some nuclear expression and was scored as positive/negative (Fig. 7). Duox expression was weakly positive in normal bone marrow, pancreas and stomach tissues; and negative in the other normal tissues evaluated, including bladder, brain, liver, lung, lymph node, small bowel and testis. The distribution of positive staining by tumor type for the multi-tumor TMA is presented in Table I. The distribution of positive staining was statistically different between tumor types by χ^2^ statistic (p<0.01). The brain tumors (glioblastoma multiforme) had the lowest rate of expression at 14%, while the prostate cancer samples had the highest frequency of expression at 92%.

## Discussion

The goal of the present study was to generate monoclonal antibodies against the human Duox2 protein to provide reagents that would be useful for investigating the role of Duox2 in cancer, where alterations in oxidant tone play a critical role in cell growth and proliferation (18). High quality, commercial monoclonal antibodies against Duox2 are not available; and thus, Duox protein expression has not been widely examined in cancers of any histology or chronic inflammatory conditions in comparison to normal tissues. Available polyclonal antibodies against human Duox2 have not been convincingly demonstrated to be reliable for most laboratory research purposes; the lack of widely available antibodies has also limited biochemical studies of this oxidase. Hence, we successfully focused our efforts on generating monoclonal antibodies against human Duox2 protein, which could be used in various immunological assays, including western blot analysis and immunohistochemistry, and that would allow a more detailed study of the physiological and pathophysiological role of Duox2 at the protein level.

Using the Duox S-12 monoclonal antibody, we confirmed our previous results demonstrating that IFN-γ upregulates Duox2 at the protein as well as mRNA levels (Fig. 2) in BxPC-3 human pancreatic cancer cells (14). More importantly, we found that in the MIA PaCa-2 human pancreatic cancer cell line, which is not responsive to IFN-γ and does not constitutively express Duox2, overexpression of Duox2 and its cognate maturation factor, DuoxA2, produced a functionally active Duox2 protein that could be quantitated with the Duox S-12 antibody (Fig. 3B). We have also clearly demonstrated the localization of Duox2 in the plasma membranes of MIA PaCa-2 cells by immunohistochemistry, and both the cytoplasmic and peripheral expression of Duox2 in these cells by immunofluorescence (Figs. 4 and 5). This distribution is similar, only in part, to that described for normal thyroid tissue (19), and respiratory and gastrointestinal epithelium (17,20) where Duox2 appears to localize at the apical surface of thyroid follicles or at the enterocyte brush border, suggesting that Duox2 is expressed only in the most differentiated of these normal cells.

As demonstrated in Fig. 6, probably as a result of the extensive amino acid homologies that exist between the two dual oxidases, we found that the Duox S-12 antibody cross-reacted with Duox1 in COS-7 cells transfected with a Duox1 cDNA. However, our recent studies have shown using real time RT-PCR that Duox1 is only minimally expressed at the mRNA level, and clearly not upregulated, in many human malignancies, including cancers of the gastrointestinal tract, breast, lung, prostate, brain and melanoma (7). Furthermore, investigators have demonstrated that expression of Duox1 is epigenetically silenced in non-small cell lung cancer (21). Thus, we felt that it was reasonable to examine human tumor tissue microarrays for expression of Duox protein with our Duox S-12 antibody under the operating assumption that we would, for the most part, be evaluating the expression and distribution of Duox2 in such experiments.

Immunohistochemical examination of Duox expression in normal human tissues and in a range of human tumors suggests that expression in carcinomas and adenocarcinomas is higher than in tumors of other histological types (melanoma, lymphoma, glioblastoma multiforme). Expression was highest in prostate adenocarcinoma and lung cancers (both adeno-carcinoma and squamous cell carcinoma); and greater than 60% in breast and colon cancer. Duox2 was expressed in only a limited number of normal tissues, none of which demonstrated strong (3+) expression, compared to the cancers, where 73% of the prostate adenocarcinomas were 3+ in expression, and 24% of breast, colon and lung cancers were 3+.

Overexpression of Duox in colon cancer is consistent with the upregulation of Duox2 mRNA that has been demonstrated in patients with two pre-cancerous conditions, Crohn’s disease and ulcerative colitis (13). The expression of Duox2 in the gastrointestinal tract has recently been demonstrated to be under the control of pro-inflammatory cytokines that are known to be associated with inflammatory bowel disease (22). Thus, the immunohistochemical studies presented here may support the hypothesis that cytokine-mediated upregulation of Duox2 could contribute to the cascade of reactive oxygen production known to accompany pre-malignant, chronic inflammatory disorders of the gastrointestinal tract (23). Furthermore, in light of recent studies demonstrating that either inhibition of Nox-related oxidant stress or other anti-inflammatory interventions can significantly diminish the late effects of gastrointestinal inflammation (24,25), our demonstration of increased Duox expression in gastrointestinal cancer suggests that pharmacologic inhibition of Nox expression might be a novel therapeutic intervention capable of interdicting the development of oxidant-mediated neoplasia.

In summary, we have developed a novel monoclonal antibody against the dual oxidase members of the Nox gene family and have used that antibody to demonstrate the over-expression of Duox2 in several human malignancies. In future studies, we will use this tool to evaluate the role of Duox2 in the development and prognosis of a variety of solid tumors, and in mechanistic studies aimed at discovering small molecule inhibitors that will specifically block the oxidase function of this protein.

## Figures and Tables

**Table I:** Distribution of expression levels of dual oxidase in human malignancies.

Tumor type (MTA-3)	Negative	Positive
Glioblastoma multiforme	12 (86%)	2 (14%)
Lymphoma	23 (79%)	6 (21%)
Melanoma	7 (64%)	4 (36%)
Ovarian Cancer	16 (55%)	13 (45%)
Breast Cancer	11 (34%)	21 (66%)
Colon Cancer	18 (38%)	29 (62%)
Lung Cancer	4 (14%)	25 (86%)
Prostate Cancer^a^	2 (8%)	24 (92%)

a Distribution of positive staining was statistically different between tumor types, p<0.01.

## Abbreviations:

Duox: dual oxidase
ROS: reactive oxygen species
Nox: NADPH oxidase
DuoxA2: dual oxidase maturation factor
